# Aerobic dichlorvos degradation by *Pseudomonas stutzeri* smk: complete pathway and implications for toxicity in *Mus musculus*

**Published:** 2020-04

**Authors:** Satish G. Parte, Ashok D. Mohekar, Arun S. Kharat

**Affiliations:** 1 Department of Zoology, M. J. S. College, Shrigonda, Ahmednagar, India; 2 Department of Zoology, Maharshi Dnyandeo Mohekar Mahavidyalaya, Kallamb, Osmanabad, India; 3 Laboratory of Applied Microbiology, School of Life Sciences, Jawaharlal Nehru University, New Delhi, India

**Keywords:** Biodegradation, *Pseudomonas stutzeri*, Fourier-transformed infrared spctroscopy, High performance liquid chromatography, Dichlorvos

## Abstract

**Background and Objectives:**

Excess use of pesticides in agricultural field not only compromised soil fertility but also posed serious threat to water bodies and life in the surrounding environment. The leftover pesticide residue needs to be remediated effectively. Compared to physical, chemical and enzymatic remediation options the microbial remediation is more practical and sustainable.

**Materials and Methods:**

The *Pseudomonas stutzeri* smk strain was found to use dichlorvos as the solitary carbon source. Minimal medium supplemented with dichlorvos was used to test ability of bacterium to degrade pesticide aerobically. The metabolites produced by the bacterium were studied with UV-Vis spectrophotometry, HPLC, FTIR and GC-MS techniques. The toxicity studies of neat dichlorvos and *P. stutzeri* smk degraded metabolites were studied by subcutaneous injection in *Mus musculus*.

**Results:**

The *P. stutzeri* smk strain was found to degrade as high as 80% of dichlorvos on 7^th^ day of incubation, at 30 °C temperature and at pH 7. In five steps complete aerobic degradation of 2,2dicholorvinyl dimethyl phosphate (dichlorvos) resulted in production of free methyl and phosphate. The degradation intermediates produced are 2-Chlorovinyl dimethyl phosphate, vinyl dimethyl phosphate, dimethyl phosphate, methylphosphate and finally free phosphate. The histopathological analysis of liver, spleen and thymus of *M. musculu*s were performed to study toxicity of dichlorvos and degraded metabolites.

**Conclusion:**

*P. stutzeri* smk could result highest aerobic degradation of dichlorvos to produce free methyl and phosphate. Degradation metabolites could reverse largely toxic effects of dichlorvos when studied in *M. musculus*.

## INTRODUCTION

The organophosphate (OP) pesticides are the group of highly toxic, heterogeneous compounds widely used for pest control. There are currently 140 OP compounds being used as pesticides and as plant growth regulators around the world (1). In agriculture, these synthetic OP compounds are widely used as insecticides. These compounds acts as acetylcholine substitutes and inhibits the acetylcholine esterase enzyme (2). In humans, OP poisoning causes various clinical effects like neck muscle weakness and diarrhea (3). Earlier reports stated that these OP compounds could cause cancer, endocrine disruption, birth defects and neurological damage (4). Among OP insecticide Dichlorvos are routinely employed insecticide for agricultural and household purposes. In view of its toxicity and persistency remediating them from the environment is highly essential. For the removal of such compounds from environment, the conventional methods like landfills, chemical treatment, incineration and recycling are employed but these methods are at times tedious, costly, time consuming and causes formation of toxin intermediates (5, 6). The most reliable and cost effective technique for pesticide removal is bioremediation, which has been successfully applied for soil contaminated with OP pesticides (7–9). Isolation of OP degrading bacteria from soil has been reported by various researchers (10–12). Some of the bacterial species are known to detoxify OP by enzymatic reactions (13). In present study we sought to address pathway of dichlorvos degradation, brought by *Pseudomonas stutzeri* smk and test toxicity of dichlorvos and its degradation metabolites by *P. stutzeri* smk in *Mus musculus.*

## MATERIALS AND METHODS

### Chemicals.

The analytical grade 95% pure dichlorvos was obtained from insecticide residue testing laboratory, Pune and all other chemicals used in current study (analytical grade and are of higher purity) were purchased from Loba chemicals, India.

### Biodegradation experiment.

The biodegradation experiments were carried as per previously described procedure (14). Instead of clothianidine the medium was supplemented with 50 μg l-1dichlorvos as a single source of carbon and phosphate and incubation time of 7 days. Leftover concentrations of dichlorvos were measured at 220 nm.

### Analytical studies.

Analytical studies for degradation were carried out by HPLC, FTIR methods previously mentioned (14). Instead LCMS analyses (14), GCMS were carried out in present study. The identification of metabolites formed after degradation was carried out using a QP2010 gas chromatography coupled with mass spectroscopy (Shimadzu). The initial column temperature was 80 °C for 2 min; then it was increased linearly from 15 °C min-1 to 210 °C, followed by a 10 °C min-1 increase up to 240 °C, where it was held for 2 min; finally there was a 5 °C min ^−1^ rise up to 280 °C, which was held for 10 min. The temperature of the injection port was 280 °C and the GC-MS interface was maintained at 280 °C. The helium carrier gas flow rate was 1.0 ml min^−1^.

### Toxicity Studies in *Mus musculus.*

Toxicity studies in *M. musculus* were carried out as per protocol described previously (14). The studies were carried out as per the CPCSEA guidelines upon the Institutional Animal Ethical Committee approval and the pesticide used in this study was dichlorvos in the place of clothianidine (14). Comparative toxicity was evaluated by injecting degradation generated metabolites and dichlorovos. The theme used to study toxicity included the histopathological analysis in the liver, spleen and thymus of *M. musculus*.

### Animals.

Five weeks old mice with mean weight 20 gram, 18 albino mice were procured for the study from institutional animal house.

### Animal housing and animal treatment schedule.

Housing of animals was carried out as per methods mentioned in Parte and Kharat (2019) for clothianidine degradation. In brief, eighteen albino mice were divided into three groups; 6 mice/ groups. One of the three groups received subcutaneous injection of dichlorovos at 1/4 ^th^ LD50 concentration, second group with 0.01 mg/kg body weight of mice while third group with placebo. After 24 h incubation mice were sacrificed by cervical dislocation method, histopathological staining of liver, thymus and spleen sections was carried out. The studies were carried out as per the CPCSEA guidelines upon Institutional Animal Ethical Committee approval.

### Histopathological studies.

Tissue samples were further processed for histopathological studies as protocol described previously (14) with a difference that clothianidine was replaced with dichlorvos.

## RESULTS

### Microorganism.

The previously isolated bacterium, *Pseudomonas stutzeri* smk that degrades clothainidine (14, 15) was used for the degradation of diclorovos pesticide.

### Biodegradation analysis.

**Fig. 1.**
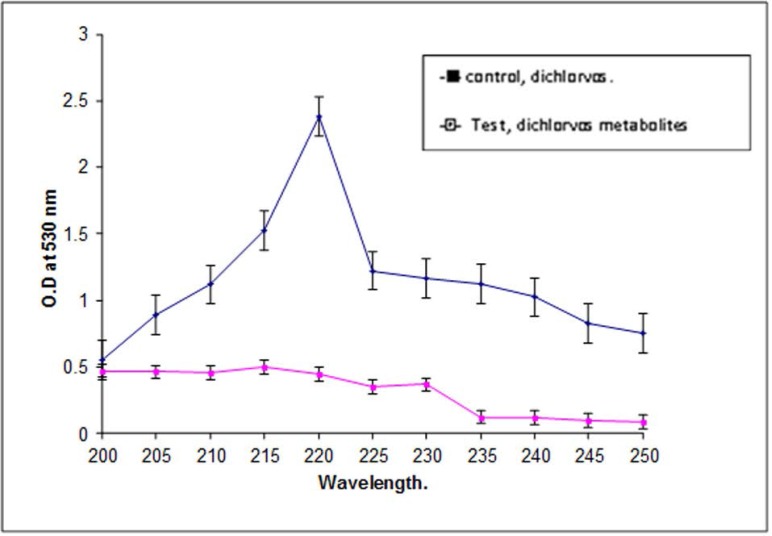
Spectroscopic analysis of dichlorvos and its degraded metabolites.

Dichlorvos was added to full-strength MSM, phosphate limiting MSM, or carbon-limiting MSM, and these media were used for the optimization of dichlorvos biodegradation. *P. stutzeri* smk showed a higher diclorvos degradation potential in full-strength MSM compared to that of phosphate or carbon-limiting MSM. For subsequent biodegradation experiments, we chose to use full-strength MSM. The percent degradation of dichlorvos was determined spectrophotometrically. Fig. 1. shows the concentration of dichlorvos measured in an extract at 220 nm following an incubation period of 7 days.

The *P. stutzeri* smk was capable of degrading as much as 80% of the total dichlorvos within a 7 days period. It is evident from Fig. 1. that the media inoculated with *P. stutzeri* smk exhibited a decline in the O.D. at 220 nm suggesting that a loss of dichlorvos occurred during the 7-day incubation period. The O.D. of the sample from the control flask, for the initial and final days remained same, suggesting that there was no intrinsic loss of dichlorvos during incubation period.

### Chromatographic and spectroscopic analysis of Dichlorvos degradation.

**Fig. 2.**
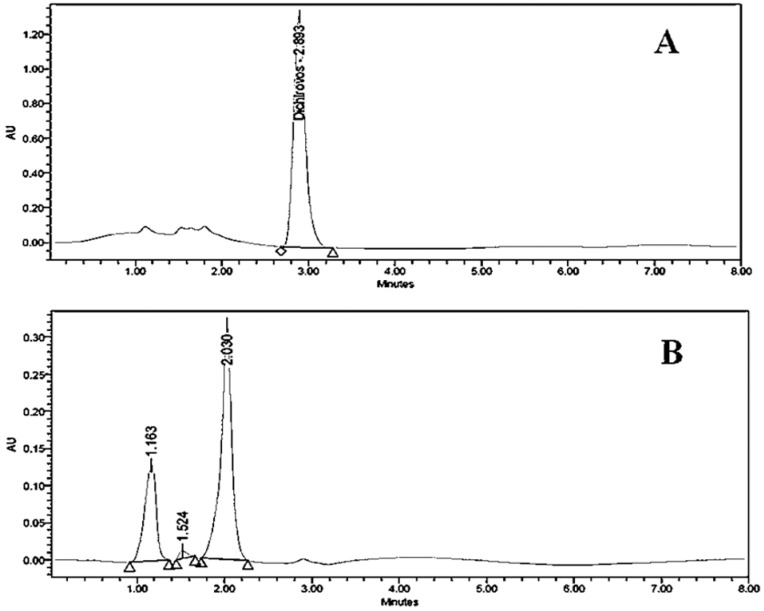
HPLC elution profile of dichlorvos standard (a) and dichlorvos biodegradation metabolites (b)

The results obtained after 7 day of incubation, showed that a reduced absorbance at 220 nm, indicating that dichlorvos was being degraded. In order to attain the highest possible dichlorvos degradation, we optimized the media and conditions then we sought to characterize the metabolites produced during degradation. The identification of metabolites was carried with chromatographic and spectroscopic analyses. The extracts from pure dichlorvos and biodegraded dichlorvos from biodegradation experiments were subjected to chromatographic comparisons of HPLC elution profiles (Methods). Fig. 2. shows comparative HPLC elution profile for pure dichlorvos and extract containing degradation metabolites after 7 days incubation.

**Fig. 3.**
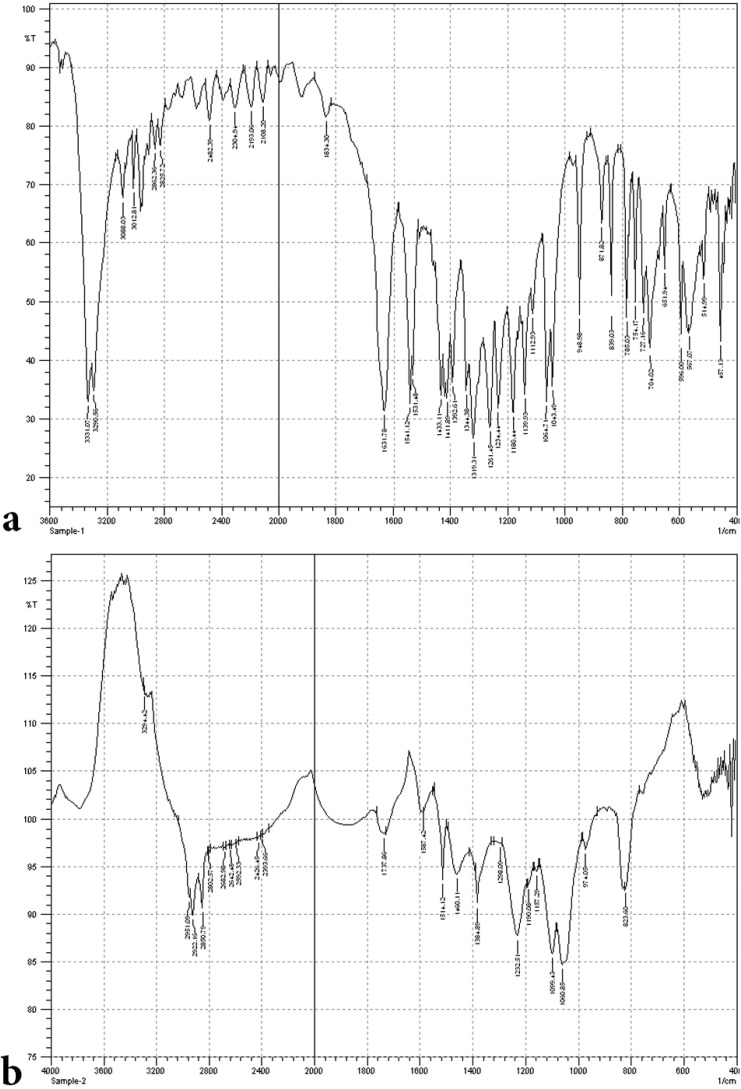
**(**a) FTIR spectrum of dichlorvos standard. (b) FTIR spectrum of dichlorvos biodegradation metabolites

It is evident from Fig. 2a that neat dichlorvos shows single peak at retention time 2.893, whereas the degradation eluate Fig. 2b exhibit three new peaks but for pure dichlorvos as well. These new peaks exhibit an altered profile with new retention time at 1.163, 1.524 and 2.030, respectively. The overall HPLC analysis of dichlorvos collectively suggests that the *P. stutzeri* smk degrades the dichlorvos into different metabolites, which was further substantiated by FTIR analysis. The FTIR analysis was performed for the confirmation of biodegradation of dichlorvos. Data shown in Fig. 3. depicts that the FTIR spectra of pure dichlorvos and its degradation metabolites were considerably different.

The FTIR spectrum for pure dichlorvos, Fig. 3a, displayed peaks at 1352.10 cm ^−1^ for P-O stretching for presence of phosphorous compound, 2958.80 cm ^−1^ for C=H stretching for vinyl group, 1149.57 cm ^−1^ , for C-O stretching for methyl group, and 659.66 cm ^−1^ for C-Cl stretching. On the other hands, the biodegradation spectra obtained with *P. stutzeri* smk, shown in Fig. 3b. exhibit peaks at 3101.54 cm ^−1^ for O-H stretching for hydroxyl group and the 1531.45 cm ^−1^ for C-H stretching of alkane group. The change in peak pattern and their position within the spectra, Figs. 3a and b, indicates that conversion of dichlorvos into new metabolite took place. Lack of peaks for O-H –hydroxyl group stretching, and C-H stretching in pure dichlorvos but in *P. stutzeri* smk degradation profile suggest degradation results in formation of new metabolites.

**Fig. 4.**
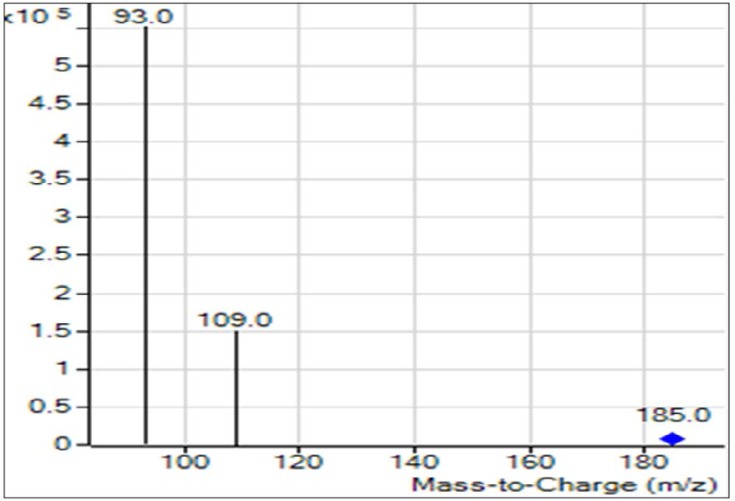
GC-MS chromatogram of major metabolites formed after diclorvos degradation.

**Fig. 5.**
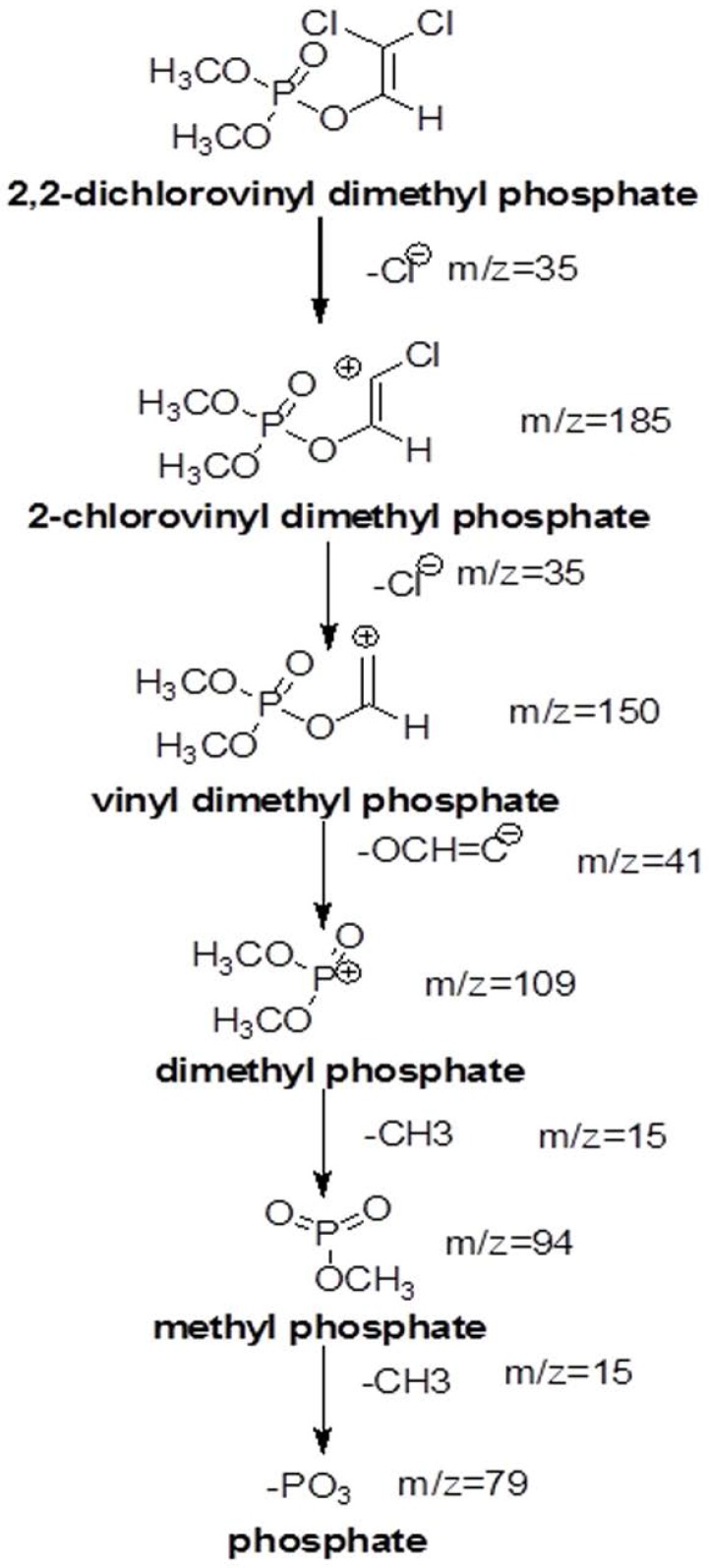
Proposed degradation pathway of Dichlorvos

The dichlorvos biodegradation pathway that we proposed on the basis of GC-MS analysis is depicted in Fig. 4. Various metabolites formed during the biodegradation of dichlorvos shown in Fig. 5, includes; 2-chlorovinyl dimethyl phosphate (m/z. 185), vinyl dimethyl phosphate (m/z 150), dimethyl phosphate (m/z 109) and phosphate (m/z 69).

### Effect of degraded metabolites on live mice.

**Fig. 6.**
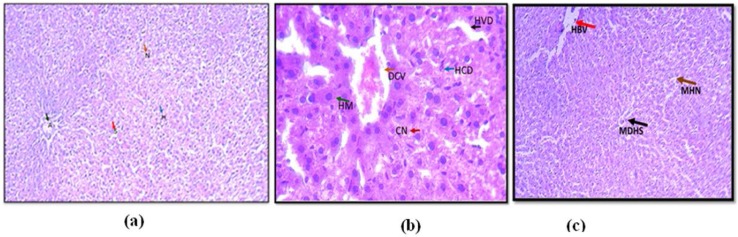
Mice were injected subcutaneously with (a) with placebo (Saline), neatdichlorvos (b) and degraded metabolites of dichlorvos (c)

Results shown in Fig. 6 demonstrate that several anomalies were noticed in the liver tissue of mice challenged with dichlorvos Fig. 6b while Fig. 6c demonstrate that most of these anomalies were reduced in liver tissue injected with degraded metabolites by *P. stutzeri* smk, and it is in large sense comparable to that of mice challenged with sterile saline (Fig. 6a).

**Fig. 7.**
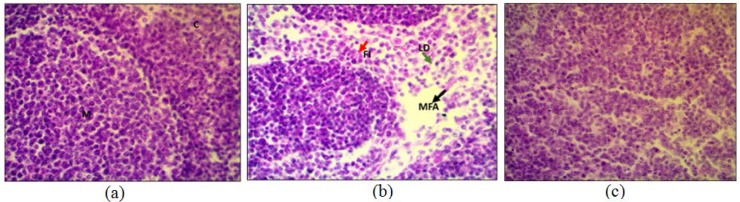
Histochemical analyses of thymus: mice we injected subcutaneously with placebo -Saline (a), neat dichlorvos (b), and degraded metabolites of dichlorvos (c).

Interestingly, anomalies were seen in the Cortex, Medulla in thymus tissue (Fig. 7b). Anomalies; lymphocytic depletion (LD), invasion of fibroblasts (FI) and focal areas of macrophoage (MFA) were also seen in histopathological analysis of thymus shown in Fig. 7b whereas most of these anomalies were significantly reduced when mice were challenged with degraded metabolites of dichlorvos, Fig. 7c. Observations were comparable to that of placebo (saline) injected (Fig. 7a).

**Fig. 8.**
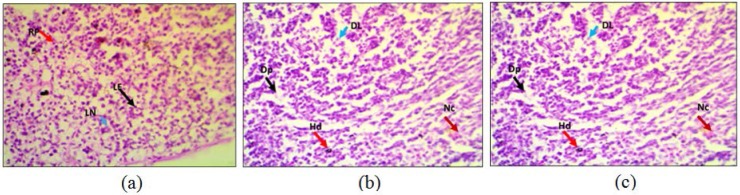
Histochemical analyses of Spleen: mice we injected subcutaneously with placebo -Saline (a), neat dichlorvos (b) and degraded metabolites of dichlorvos (c).

The lymphatic nodules (LN) of white pulp, splenic cord of red pulp (RP) and unaltered lymphoid follicles (LF) were seen in histopathological studies of spleen in mice challenged with saline (Fig. 8a). Curiously mice challenged with neat dichlorvos spleen tissue exhibited disorganized lymphocyties (DL) in lymphaid follicles, hemosiderin deposition (Hd), depopulation (Dp) and necrosis (Nc) (Fig. 8b). While mice challenged with degraded metabolites of dichlorvos spleen tissue exhibit only moderate anomalies (Fig. 8c) suggesting that *P. stutzeri* SMK mediated degradation was a great success in terms of reducing toxicity of dichlorvos.

## DISCUSSION

Dichlorvos (DDVP, 2, 2-dichlorovynil dimethyl phosphate) is an organophosphorous pesticide with high solubility in water and largely used for agricultural purposes. The dichlorvos acts by inhibiting acetylcholine esterase, an enzyme that is very important in the nervous system of all vertebrates and some invertebrates. The present study endeavor was bacterial degradation of dichlorvos with *P. stutzeri* smk and to evaluate the toxicity of their dichlorvos versus degradation metabolites formed in *Mus musculus.* Here we report that the species is *P. stutzeri* smk that degraded the clothainidine pesticides (14) could also degrade as high as 80% of dichlorvos within 7 days, to the best of our knowledge this is fastest and highest rate of bacterium mediated degradation of dichlorvos. Jiang et al. (16), reported that strain *Roseomonas* sp. was able to degrade 69.0% of dichlorvos. Recently Agarry et al. (17) reported that the strain *Proteus vulgaris* showed the highest removal rate, 70%. Till date a number of bacterial species have been reported for dichlorvos degradation. Recently, the *Flavobacterium* YD4 strain reported for degradation of the dichlorvos (18). Several microorganisms that are capable of degrading dichlorvos have been reported; these include *Pseudomonas* spp. *Proteus vulgaris, Vibrio* spp., *Serratia* spp. and *Acinetobacter* spp. (17–18). The halophiles, *Halomonas* spp. (19) and *Nocardia mediterranei* (20) are also reported to degrade dichlorvos.

The optimization of physiochemical parameters for biodegradation of dichlorvos revealed that pH 7 and the temperature 30 °C was optimum for degradation at which maximum degradation of 80% was attained. It was reported that optimization of pH and temperature could positively influence bioremediation rate (14).

Deshpande et al. (21) studied the degradation of OP compound, dimethoate by using *Brevundimonas* spp. MCM B-427 and reported that the optimization of temperature and pH caused significant increase in biodegradation of dimethoate (21). In another studies plasmid mediated dimethoate degradation was studied, the trait was found to be transferable in other bacteria by means of genetic recombination (22) Studies on acephate biodegradation by bacterial consortium of *Exiguobacterium* sp. BCH4 and *Rhodococcus* sp. BCH2 have been reported earlier also showed effect of temperature and pH optimized degradation (23). Studies with the increasing dichlorvos concentration revealed that the rate of dichlorvos degradation was reduced upon increasing dichlorvos concentration (18). We reported in our study that at lower concentration of dichlorvos it supported bacterial growth while increased concentration might be toxic to the bacterium. The cells and enzyme system might have got hampered by increasing concentration of pesticide, hence it results into reduced biodegradation. In 2012, it is reported that increased concentration of dichlorvos hampers the rate of degradation by *Flavobacterium* YD4 (18). The UV-Vis spectroscopic analysis reveals that the biodegradation of dichlorvos. The HPLC analysis of dichlorvos before and after microbial treatment confirmed the biodegradation of dichlorvos into different metabolites. The observations obtained through UV-vis and HPLC analysis are supported by FTIR results. The dichlorvos degradation pathway by *P. stutzeriv* smk was deduced on the basis of GC-MS analysis. The chromatograms of degraded dichlorvos showed the presence of three peaks and the structures of the detected compounds were assigned from the fragmentation pattern and m/z values obtained. From present study, it was observed that the dichlorvos first underwent dechlorination reaction to form 2-chlorovinyl dimethyl phosphate (m/z. 185) which by subsequent dechlorination produced vinyl dimethyl phosphate (m/z 150). The vinyl dimethyl phosphate underwent asymmetrical cleavage to form dimethyl phosphate (m/z 109). Finally, the dimethyl phosphate undergoes two successive demethylation steps resulting into release of phosphate, which is further utilized by the bacteria as a sole source of phosphorous. Dichlorvos pesticide is toxic to animals and humans (24). In present study we evaluated the toxicity of dichlorvos and their degraded intermediates by analyzing histo-pathological changes in the liver, spleen, and thymus of the *Mus musculus*. The liver is the principle detoxifying organ, so, it widely exposed to a variety of external and internal products like environmental toxins and chemicals present in food or drinking (14). In this study, the histopathological analysis of liver of mice, treated with dichlorvos demonstrated several histopathological alterations. Hepatic cells necrosis with diffuse vacuolar degeneration of hepatocytes was observed. Sharma and Singh (24) studied the hepatotoxicity and reported that, the reactive oxygen species (ROS) generated due to dichlorvos toxicity leads to degeneration of hepatocytes and their necrosis (24, 25). Central veins and other hepatic blood vessels were dilated shown in Fig. 6b. Similar results were obtained by El-bendary (26) upon treatment of chlorpyriphos to *Mus musculus* (26). Fig. 6c showed the liver of mice treated with degraded metabolite of dichlorvos, which showed mild dilation of hepatic sinusoid and hepatic blood vessels as compared to mice liver treated with dichlorvos. There was mild hepatic necrosis observed when mice were treated with degradation metabolites in comparison with neat dichlorvos treated mice liver. There was no any alteration in the liver architecture when mice were placebo treated (Fig. 6a). The organ of interest for histopathological study was thymus. Histopathology analysis of the thymus of mice challenged with neat dichlorvos (Fig. 7b) exhibited abnormalities in normal architecture of cortex and medulla. The lymphocytic depletion, invasion of fibroblasts and focal areas of macrophage activity were observed. Whereas mice treated with degraded metabolites by *P. stutzeri* smk showed only mild abnormalities in normal architecture of cortex and medulla (Fig. 7c). The thymocyte hypertrophy was seen and rare focal areas of macrophage activity were observed. Our results are in agreement with results obtained in toxicity study of Profenofos and Chlorpyrifos in mice (26).

Histopathology analysis carried on the spleen of mice challenged with neat dichlorvos showed change in the normal architecture of mice spleen after treating with dichlorvos (Fig. 8b). The disorganization of lymphocytes in lymphoid follicles, hemosiderin deposition, depopulation, necrosis and vascular changes were observed. When mice were treated with degraded metabolites it showed a few alterations in the spleen histology (Fig. 8c). These were mild alterations in the architecture as compared to the dichlorvos treated mice. Moderate increase in the hemosiderin and necrosis were observed, however these were significantly reduced to that of neat dichlorvos treated mice.

The present study provides valuable information regarding the dichlorvos biodegradation potential of *Pseudomonas stutzeri* smk and the study was carried out with respect to the toxicity of dichlorvos and their degraded metabolites showing significant reduction in the toxicity level upon treatment of *Pseudomonas stutzeri* smk. To the best of our knowledge *P. stutzeri* smk strain degraded fastest and highest amount of dichlorvos that the earlier bacteria known to degrade. This article elucidates aerobic degradation pathway of dichlorvos (2,2dichlorovinyl dimethyl phosphate) by *P. stutzeri* smk; two dechlorination steps producing 2chlorovinyl dimethyl phosphate and vinyl dimethyl phosphate respectively. The vinyl dimethyl phosphate was then devinylated to produce dimethyl phosphate which upon two sequential demethylation steps was able to separate 2-methyl moieties and a free phosphate to serve it as the sole carbon and phosphate source to support growth.

## References

[1] Kang DG Choi SS Cha HJ. Enhanced biodegradation of toxic organophosphate compounds using recombinant *Escherichia coli* with sec pathway-driven periplasmic secretion of organophosphorus hydrolase. Biotechnol Prog 2006; 22: 406–410. 16599554 10.1021/bp050356k

[2] Bakry NM el-Rashidy AH Eldefrawi AT Eldefrawi ME. Direct actions of organophosphate anticholinesterases on nicotinic and muscarinic acetylcholinic receptors. J Biochem Toxicol 1988; 3: 235–259. 3236334 10.1002/jbt.2570030404

[3] Grimsley J Rastogi V Wild J. Biological detoxification of organophosphorus neurotoxins. In: Bioremediation Principles and Practice-Biodegradation Technology Developments (Sikdar S. Irvine R., Eds). Technomic Publishers, New York 1998; pp 557–613

[4] Miller WR Sharpe RM. Environmental estrogens and human reproductive cancers. Endocr-Relat Cancer 1998; 5: 69–96.

[5] Dua M Singh A Sethunathan N Johri AK. Biotechnology and bioremediation successes and limitations. Appl Microbiol Biotechnol 2002; 59: 143–152. 12111139 10.1007/s00253-002-1024-6

[6] Richins RD Kanera I Mulchandani A Chen W. Biodegradation of organophosphorus pesticides by surface-expressed organophosphorus hydrolase. Nat Biotechnol 1997; 15: 984–987. 9335050 10.1038/nbt1097-984

[7] Singh BK Walker A Wright DJ. Degradation of chlorpyrifos, fenamiphos, and chlorothalonil alone and in combination and their effects on soil microbial activity. Environ Toxicol Chem 2002; 21: 2600–2605. 12463554

[8] Yang L Zhao Y Zhang B Yang C Zhang X. Isolation and characterization of a chlorpyrifos and 3,5,6-trichloro-2-pyridinol degrading bacterium. FEMS Microbiol Lett 2005; 251: 67–73. 16143458 10.1016/j.femsle.2005.07.031

[9] Lakshmi CV Kumar M Khanna S. Biodegradation of chlorpyrifos in soil by enriched cultures. Curr Microbiol 2009; 58: 35–38. 18815830 10.1007/s00284-008-9262-1

[10] Zhongli C Shunpeng L Guoping F. Isolation of methyl parathion-degrading strain M6 and cloning of the methyl parathion hydrolase gene. Appl Environ Microbiol 2001; 67: 4922–4925. 11571204 10.1128/AEM.67.10.4922-4925.2001 PMC93251

[11] Chang TC Chang HC Yu FW Lenie D Mario V Chung TT. Species-level identification of isolates of the *Acinetobacter* calcoaceticus-*Acinetobacter baumannii* complex by sequence analysis of the 16S–23S rRNA gene spacer region. J Clin Microbiol 2005; 43:1632–1639. 15814977 10.1128/JCM.43.4.1632-1639.2005 PMC1081347

[12] Horne I Harcourt RL Sutherland TD Russel RJ Oakeshott JG. Isolation of a *Pseudomonas monteili* strain with a novel phosphotriesterase. FEMS Microbiol Lett 2002; 206: 51–55. 11786256 10.1111/j.1574-6968.2002.tb10985.x

[13] Huang J Qiyo CL Li X Xing JM. Cloning and fusion expression of detoxifying gene in *Escherichia coli*. Acta Genetica Sinica 2001; 28:583–588. 11431993

[14] Parte SG Kharat AS. Aerobic degradation of clothianidin to 2-chloro-methyl thiazole and methyl 3-(thiazole-yl) methyl guanidine produced by *Pseudomonas stutzeri* smk. J Environ Public Health 2019; 2019:4807913. 30944570 10.1155/2019/4807913 PMC6421824

[15] Parte SG Kharat AS Mohekar AD. Isolation and characterization of dichlorvos degrading bacterial strain *Pseudomonas stutzeri* smk. RJLBPCS 2017; 2: 282–288.

[16] Jiang Y Deng Y Liu X Xie B Hu F. Isolation and identification of a bacterial strain JS018 capable of degrading several kinds of organophosphate pesticides. Wei Sheng Wu Xue Bao 2006; 46: 463–466. 16933622

[17] Agarry SE Olu-Arotiowa OA Aremu MO Jimoda LA. Biodegradation of Dichlorovos (Organophosphate pesticide) in soil by bacterial isolates. J Nat Sci Res 2013; 3:12–17.

[18] Ning J Gang G Bai Z Hu Q Qi H Ma A In situ enhanced bioremediation dichlorvos by a phyllosphere *Flavobacterium* strain. Front Env Sci Eng 2012; 6:231–237.

[19] Oncescu T Oancea P Enache M Popescu G Dumitru L Kamekura M. Halophilic bacteria are able to decontaminate dichlorvos, a pesticide, from saline environments. Cent Eur J Biol 2007; 2:563–573.

[20] Sukirtha TH Usharani MV. Production and qualitative analysis of biosurfactant and biodegradation of the organophosphate by *Nocardia mediterranie*. J Bioremed Biodeg 2013; 4: 198.

[21] Deshpande NM Sarnaik SS Paranjpe SA Kanekar PP. Optimization of dimethoate degradation by *Brevundimonas* sp. MCM B-427 using factorial design: studies on interactive effects of environmental factors. World J Microb Biotechnol 2004; 20: 455–462.

[22] Deshpande NM Dhakephalkar PK Kanekar PP. Plasmid-mediated dimethoate degradation in *Pseudomonas aeruginosa* strain MCMB-427. Lett Appl Microbiol 2001; 33:275–279. 11559400 10.1046/j.1472-765x.2001.00995.x

[23] Phugare SS Gaikwad YB Jadhav JP. Biodegradation of acephate using a developed bacterial consortium and toxicological analysis using earthworms *(Lumbricusterrestris)* as a model animal. Int Biodeter Biodegr 2012; 69: 1–9.

[24] Sharma P Singh R. Dichlorvos and Lindane induced oxidative stress in rat brain: Protective effects of ginger. Pharmacognosy Res 2012; 4: 27–32. 22224058 10.4103/0974-8490.91031 PMC3250036

[25] Owoeye O Edem FV Akinyoola BS Rahaman S Akang EE Arinola GO. Histological changes in liver and lungs of rats exposed to dichlorvos before and after vitamin supplementation. Eur J Anat 2012; 16: 190–198.

[26] El-bendary HM Shaker MH Saleh AA Negm SE Khadey ME Hosam Eldeen FA. Histopathological changes associated with exposure of male mice to profenofos and chlorpyrifos. Annu Res Rev Biol 2014; 4: 766–777.

